# Skill retention after school-based CPR training – a systematic review and meta-analysis

**DOI:** 10.1186/s12889-025-25024-w

**Published:** 2025-11-05

**Authors:** Umut Onbasilar, Thilo von Groote, Rebecca Brülle, Hendrik Booke, Ludwig Maximilian Schöne, Christian Strauß, Hugo Van Aken, Antje Gottschalk, Mahan Sadjadi

**Affiliations:** 1 Department of Anaesthesiology, Intensive Care and Pain Medicine, University Hospital Münster, Albert-Schweitzer-Campus 1, Münster, 48149 Germany; 2 Department of Anaesthesiology, Intensive Care and Pain Medicine, Florence Nightingale Hospital, Kreuzbergstraße 79, Düsseldorf, 40489 Germany

**Keywords:** CPR training, School-based, Skill retention, Systematic review, Meta-analysis

## Abstract

**Background:**

Early cardiopulmonary resuscitation (CPR) is a key intervention for improving outcomes after out-of-hospital cardiac arrest. School-based CPR training is aimed at enhancing bystander CPR rates. However, uncertainty exists regarding long-term retention of CPR skills and the need for refresher training. This systematic review and meta-analysis assessed the effectiveness of school-based CPR training regarding skill acquisition and retention.

**Methods:**

A pre-registered (CRD42021249778) systematic search across eight databases identified randomized controlled trials (RCTs) and non-randomized studies of interventions (NRSIs) evaluating school-based CPR training (in participants aged 6–18 years) with follow-up assessments after ≥ 3 months. Key outcomes were correct technique and skill retention over time. Study quality was assessed systematically using the ROBINS-I and RoB 2 tools. Meta-analysis of weighted means and paired comparisons was performed.

**Results:**

Eighteen studies (twelve RCTs and six NRSIs) met the inclusion criteria. CPR training was associated with a significant improvement in skill acquisition, with rates of correct CPR performance ranging from 74% to 90% across various components. However, a notable decline in skill retention was observed over time, with a particularly marked reduction in chest compression quality within 6 to 8 months post-training.

**Conclusion:**

School-based CPR training effectively teaches children CPR skills, but these newly acquired skills may deteriorate over time, suggesting a need for periodic refresher courses to sustain proficiency. Standardized evaluation methods and reinforcement strategies could be incorporated into training programs in an effort to ensure sustained competence and maximize educational outcomes.

**Supplementary Information:**

The online version contains supplementary material available at 10.1186/s12889-025-25024-w.

## Background

Early cardiopulmonary resuscitation (CPR) is a key intervention to improve outcomes of individuals experiencing out-of-hospital cardiac arrest, a leading cause of morbidity and mortality worldwide [1, 2]. Out-of-hospital cardiac arrest has an annual incidence of 30–97 cases per 100,000 individuals and low survival rates, generally below 10% [3]. Survival and patient-centered outcomes are heavily influenced by the timely initiation of CPR and defibrillation, and early intervention by bystanders significantly increases chances of survival [4].

The “chain of survival” concept emphasizes a coordinated response to cardiac arrest situations, including early recognition, activation of emergency services, immediate (bystander) CPR, and early defibrillation [5]. Both the American Heart Association and the European Resuscitation Council regularly update guidelines to reflect evidence-based practices, with an emphasis on early start of high-quality chest compressions, including achieving an optimal rate and depth, full chest recoil, and minimizing interruptions to maximize efficacy [2, 6]. The increasing availability of automated external defibrillators in public spaces has further increased survival likelihood by enabling timely defibrillation [1, 2, 7, 8]. Enhancing bystander CPR rates is a major opportunity to improve outcomes. One of the most promising strategies to achieve this is simulation-based training [6, 7]. Educational institutions, particularly schools, are ideal settings for implementing this type of training. Early exposure to CPR training may increase bystander CPR rates [8–10]. Beyond the effectiveness of school-based training, the retention of CPR skills over time is a critical concern as knowledge about retention rates is essential to determine the need for periodic refresher trainings. This systematic review and meta-analysis evaluates the effectiveness of school-based CPR training in improving CPR knowledge and skills among school-aged children (6–18 years) over time, and examines skill retention rates, by analyzing data from both randomized controlled trials (RCTs) and non-randomized studies of interventions (NRSIs).

## Methods

### Study design and search strategy

A protocol for this review was submitted to the International Prospective Register of Systematic Reviews (PROSPERO) before initiation of the review procedures (CRD42021249778). PRISMA guidelines were followed in the study conduct and reporting [11]. No ethical approval was required since the analysis involved exclusively publicly available data.

An initial search was conducted in April 2021 across the following databases: MEDLINE (Ovid), CINAHL (EBSCO), SCI-EXPANDED, SSCI, A&HCI, CPI-SSH, ESCI (Web of Science), and CENTRAL (Cochrane Library). The search strategy was constructed by combining keywords and MeSH terms for the core components of the research questions: terms for the population and setting were combined with terms for the intervention. (Supplementary File 1). The search was updated in March 2023 and July 2025. No restrictions were placed on publication type, date or language. Retrieved records were imported into Zotero 5.0, and duplicates removed. Titles and abstracts were screened against the inclusion and exclusion criteria, and full texts were reviewed by two authors independently (UO and MS). Data were extracted using a standardized form. Discrepancies in extracted data were resolved through consensus between authors.

### Eligibility criteria

Studies were eligible for inclusion if they met the following criteria:

- Population (P): School-aged children, 6–18 years
- Intervention (I): School-based CPR training programs
- Comparison (C): Standard first aid training or alternative training methods
- Outcomes (O): CPR knowledge, practical skills retention, willingness to perform CPR, time to call EMS or actual resuscitation performance
- Timing (T): Long-term follow-up of ≥ 3 months to assess skill retention and knowledge maintenance
- Setting (S): School-based CPR trainings aimed at students, evaluated in either RCTs or NRSIs

### Outcomes

The following outcomes were analyzed:

- Percentage of participants who called emergency medical services (EMS)
- Percentage of participants demonstrating correct hand positioning
- Percentage of participants demonstrating correct chest compression depth
- Percentage of participants demonstrating correct chest compression rate
- Mean chest compression depth
- Mean chest compression rate

### Quality assessment

The risk of bias for each study was independently assessed by two authors (UO and MS). For non-randomized studies, the Risk of Bias in Non-Randomized Studies of Interventions (ROBINS-I) tool was used [12]. For randomized controlled trials, the Cochrane risk-of-bias tool for randomized trials (RoB 2) was used [13].

### Statistical analysis

The approach to statistical (meta-)analysis was designed to account for substantial heterogeneity across the included studies. For continuous outcomes, pooled weighted means were calculated at at predefined follow-up time points (2–4 Months and 6–8 Months).

Meta-analysis was conducted where relevant data were available, and pooled effect sizes and 95% confidence intervals (CIs) were calculated with the Mantel-Haenszel method under the assumption that the effects across studies were similar. Both fixed-effects and random-effects models were applied, and the random-effects model was generally preferred when significant heterogeneity was detected. We assessed heterogeneity across studies with the I² statistic and Cochran’s Q test. An I² value > 50% was considered to indicate substantial heterogeneity.

To assess time-sensitive changes in CPR skill retention, we used paired t-tests to compare the weighted means at different follow-up time points (e.g. 3 and 6 months). Statistically significant differences in these comparisons were interpreted as suggestive of skill decay (or improvement) over time. P values and significance levels should be considered as exploratory.

The risk of bias for any examined outcome was assessed with ROBINS-I or RoB 2, as outlined above. Due to the nature of the topic, and the results of studies to date, publication bias was not considered a major concern and was not specifically assessed in the analysis. Statistical analyses (including weighted means, paired t-tests, and meta-analyses) and data visualization were conducted in R software (R Foundation for Statistical Computing, Vienna, Austria).

## Results

**Fig. 1:**
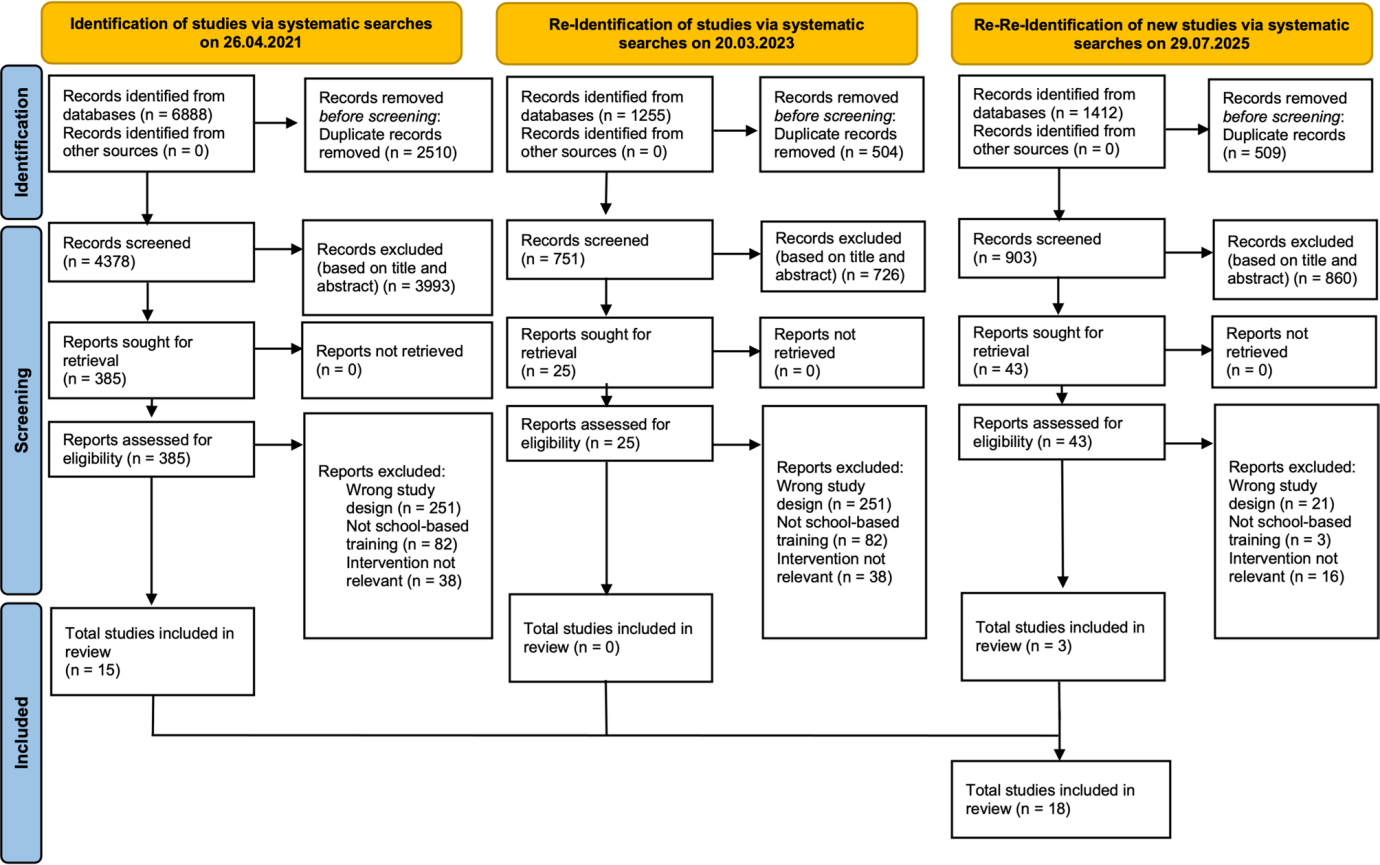
PRISMA flowchart

A systematic literature search yielded at total of 9555 records. After removal of 3523 duplicates, 6032 titles and abstracts were screened. Full texts were retrieved for 453 articles, of which 435 studies were excluded for reasons including unsuitable study design, lack of school-based training focus, or unavailability of data. In total, 18 studies (12 RCTs and 6 NRSIs) were included in the meta-analysis (Fig. 1).

### Characteristics of the included studies

#### Randomized controlled trials (RCTs)

The review incorporated twelve RCTs, involving a total of 3665 participants from various countries, predominantly in primary and secondary school settings. The sample sizes of these studies ranged from 36 to 1124 participants. The studies had diverse designs, intervention strategies, and follow-up durations. These studies collectively reflected the variability in implementation of CPR training programs across educational settings (Table 1).

**Table 1:** Descriptive characteristics of RCTs

Study	Country	Intervention Details	Control Group	Intervention Group	Outcome Measures	Outcome Time Points
Beskind et al., 2016 [14]	USA	(Sham video) vs. brief video and chest-compression-only (CCO) CPR training	Sham video	CCO CPR training via brief video and classroom training	Chest compression quality and responsiveness to emergencies	Baseline, post-intervention, and after 2 months
Cuijpers et al., 2016 [15]	Netherlands	CPR training by a registered nurse / medical student vs. physical education teacher	Physical education teacher-led training	Nurse-led CPR training	CPR skill acquisition and performance	Post-intervention and after 2 months
del Pozo et al., 2014 [16]	Spain	CPR training vs. additional video/song summarizing CPR safety checks	CPR training course only	CPR course and video/song summary	Knowledge of CPR steps and chest compression accuracy	Baseline, post-intervention, and after 8 months
Nord et al., 2016 [17]	Sweden	CPR training delivered via DVD vs. mobile app	DVD-based training	App-based training	Cardiff test score and willingness to perform CPR	Post-intervention and after 6 months
Nord et al., 2017 [18]	Sweden	CPR training with or without an online course	Standard CPR training	Web course + CPR training	CPR skill retention	Post-intervention and after 6 months
Sabihah et al., 2020 [19]	Malaysia	Peer-led CPR training vs. basic life support trainer-led training	Peer-led CPR training	Trainer-led CPR training	Psychomotor skills in performing CPR	Baseline, post-intervention, and after 3 months
Van Raemdonck et al., 2014 [20]	Belgium	Teacher-led CPR with manikin vs. peer-led training with foam dice/plastic bag + video instruction	Teacher-led CPR with manikin	Peer-led CPR with video	CPR skill proficiency	Post-intervention and after 6 months
Yeung et al., 2017 [21]	United Kingdom	Lifesaver app-based training, face-to-face training (F2F), or a combination of both	Standard F2F BLS training	Lifesaver app training	Skill retention and knowledge gain	Post-intervention and after 3 and 6 months
Chamdawala et al., 2021[22]	USA	Inflatable manikin training vs. real-time visual feedback device for CPR	Inflatable manikin practice	Real-time feedback-based CPR training	Chest compression accuracy and depth	Post-intervention, week 10, 28, and 52
Isa MH et al., 2019 [23]	Malaysia	CPR training by medical students vs. teachers	Teacher-led training	Medical student-led training	Retention of CPR knowledge and skills	Post-intervention and after 3 months
Spartinou et al., 2024 [24]	Greece	CPR training by healthcare professionals vs. teachers vs. student peers	Teacher- or healthcare-professional-led training	Peer-led training	Effectiveness of schoolchildren as peer instructors for CPR training and compliance with the CPR algorithm	Post-intervention and after 6 months
Yeung et al., 2023 [25]	Hong Kong	CPR training by teachers vs. healthcare instructors	Teacher instructor	Healthcare instructor	Passing rate of students’ BLS performance skills at 6-month follow-up and changes in the knowledge and attitudes	Post-intervention and after 6 months

#### Non-randomized studies of interventions

Six NRSIs encompassing 3715 participants were included. The sample sizes for these studies ranged from 30 to 2579 participants. These studies were heterogeneous in their scope and methodological approaches, and combined theoretical education with practical skills, often incorporating interactive training tools to enhance learning (Table 2).

**Table 2:** Descriptive characteristics of NRSIs

Study	Country	Design	Intervention Details	Outcome Measures	Outcome Time Points
Meissner et al., 2012 [26]	Germany	Two-hour training course	Theoretical background on sudden cardiac death and hands-on CPR tutorial	Knowledge and performance of BLS before and after training	Post-training and after 2-4 months
Banfai et al., 2017 [27]	Hungary	Three-session course	45-minute sessions over 3 weeks covering theoretical knowledge and practical skills in first aid	Knowledge improvement and skill retention	Post-training and after 2-4 months
Dhansura et al., 2020 [28]	India	One-hour training session	Compression-only life support (COLS) CPR with hands-on practice	Feasibility of teaching COLS and understanding of content	Post-training and after 2-4 months
Naqvi et al., 2011 [29]	Pakistan	5-hour course	DVD-based resuscitation course with demonstrations and hands-on practice	Knowledge and skill retention measured	Post-training and after 2-4 months
Müller et al., 2014 [30]	Germany	90-minute training including video support	Video-supported CPR training	Knowledge Retention	Post-training and after 8 months
Cons-Ferreiro et al. 2023 [31]	Spain	50-min theoretical-practical course and virtual classroom	Control group (traditional training programme) and experimental group (flipped classroom training programme)	Medium and long-term retention of knowledge and practical skills	Post-training, after 6 months and after 12 months

### Risk of bias in the included studies

#### Randomized controlled trials

**Fig. 2:**
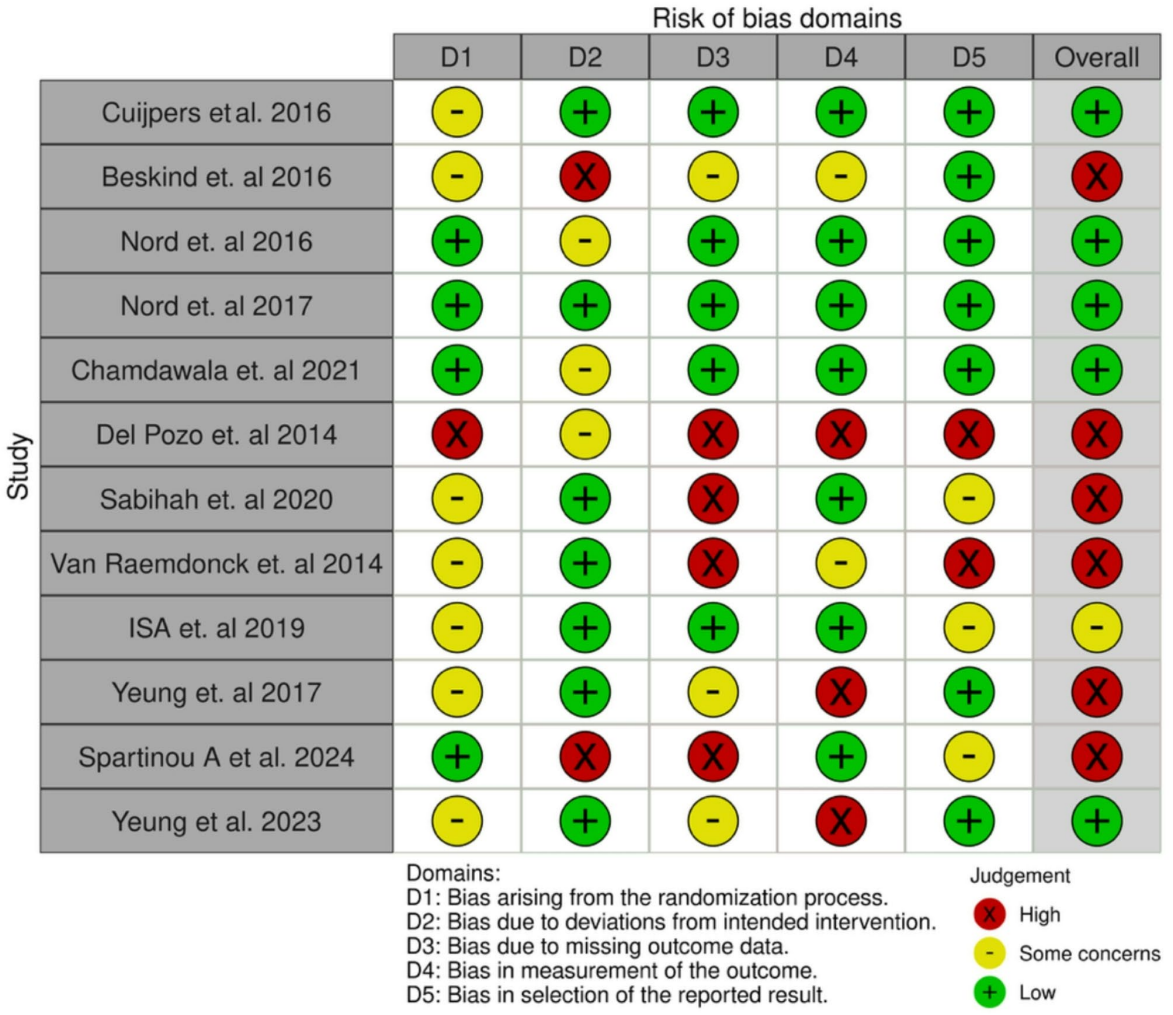
Risk of bias summary for RCTs

The quality of the included RCTs was heterogenous. Six studies had high risk, three raised some concerns and three had low risk. The most common issues resulting in an increased risk of bias were missing outcome data and deviations from intended interventions (Fig. 2).

#### Non-randomized studies of interventions

**Fig. 3:**
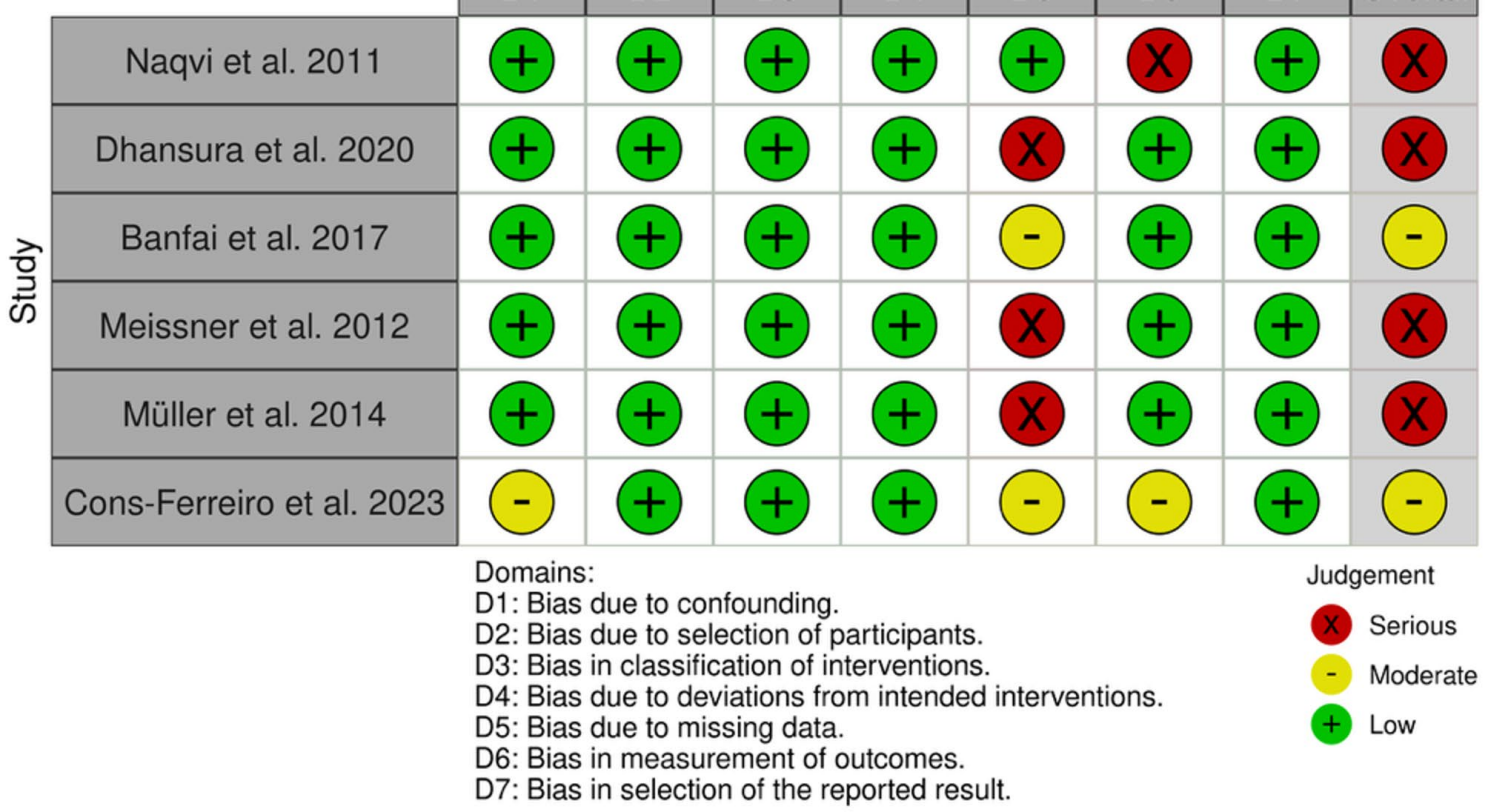
Risk of bias summary for NRSIs

The overall risk of bias for the included NRSIs was moderate to serious, with serious concerns identified in four studies. The most consistent source of bias across all studies was missing data. Fewer concerns existed for confounding, selection bias and classification of interventions (Fig. 3).

### Outcomes of interest

#### Calling emergency medicine services

##### Overall quality of data

The included RCTs assessing the outcome of calling emergency medical services exhibited varying levels of bias [14, 23]. Two studies [19, 24] with longer follow-up periods (6–8 months) exhibited high risk of bias [19, 24] due to deviations from the intended intervention and missing outcome data. Two other contributing studies [17, 18] showed low risk of bias.

Among the NRSIs, the papers by Dhansura et al. [28] and Meissner et al. [26] had some risk of bias because of missing data. Additional concerns associated with outcome measurement existed for the report by Naqvi et al. [29].

##### Changes in outcome accomplishment over time

**Fig. 4:**
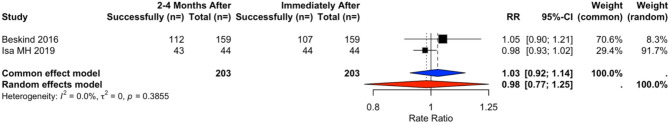
Rate ratio analysis of calling EMS in RCTs immediately after vs. 2–4 months after training

**Fig. 5:**
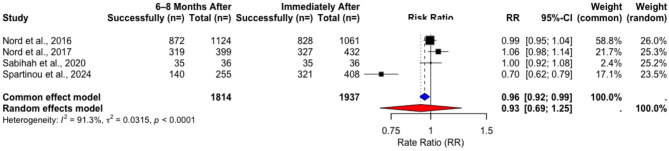
Rate ratio analysis of calling EMS in RCTs immediately after vs. 6–8 months after training

The pooled weighted performance success rates for “Calling emergency medicine services” did not change significantly over time (Tables 3 and 4) in the included RCTs. Rate ratio (RR) analysis found no significant difference in success rates after two to four months (Common-Effect RR = 1.03, 95% CI [0.9222, 1.1423], *p* = 0.633), with no evidence of heterogeneity (Fig. 4). The analysis of long-term (6–8 months) outcomes revealed some hetereogeneity, so a random effects model was used in the analysis, again revealing no statistically significant difference in success rates (Random-Effects RR: 0.93, 95% CI [0.69, 1.25], *p* = 0.4938) (Fig. 5).

**Table 3:** Changes in rates of calling EMS in RCTs immediately after vs. 2–4 months after training

Study	Immediately After		2–4 Months After		t	*p*
	Total Count (*n*=)	Performance Success Rate (%)	Total Count (*n*=)	Performance Success Rate (%)		
Beskind et al. 2016 [14]	159	67,3	159	70,4		
Isa MH et al. 2019 [23]	44	100	44	97,7		
Pooled Weighted Results	**203**	**74**,**39**	**203**	**76**,**32**	**−0.65932**	**0.6289**

**Table 4:** Changes in rates of calling EMS in RCTs immediately after vs. 6–8 months after training

Study	Immediately After		6–8 Months After		t	*p*
	Total Count (*n*=)	Performance Success Rate (%)	Total Count (*n*=)	Performance Success Rate (%)		
Nord et al. 2016 [17]	1061	78,04	1124	77,58		
Nord et al. 2017 [18]	432	75,69	399	79,95		
Sabihah et al. 2020 [19]	36	97,22	36	97,22		
Spartinou et al. 2024 [24]	408	78,67	255	54,9		
Pooled Weighted Results	**1937**	**78**,**01**	**1814**	**75**,**30**	**0.228**	**0.8343**

**Fig. 6:**
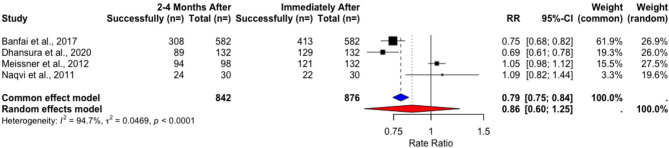
Rate ratio analysis of calling EMS in NRSIs immediately after vs. 2–4 months after training

**Fig. 7:**
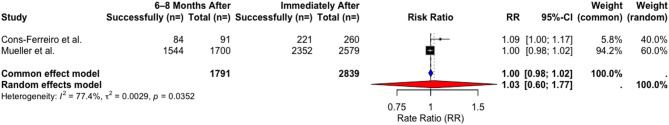
Rate ratio analysis of calling EMS in NRSIs immediately after vs. 6–8 months after training

In non-randomized studies, the weighted performance rates decreased from 78.25% to 61.16% 2–4 months after training (t = 1.8589, *p* = 0.16) (Table 5), and the change after 6–8 months was virtually non-existent, from 90,63% to 90,88% (t = −0.03793, *p* = 0.9759) (Table 6). A Rate Ratio analysis accounting for heterogeneity using a random effects model showed no statistically significant difference (RR = 0.8642, 95% CI [0.6899, 1.0826], *p* = 0.2043 and RR = 1.0310, 95% CI [0.6014; 1.7675], *p* = 0.6029) (Figs. 6 and 7).

**Table 5:** Changes in rates of calling EMS in NRSIs immediately after vs. 2–4 months after training

Study	Immediately After		2–4 Months After			t	*p*
	Total Count (*n*=)	Performance Success Rate (%)	Total Count (*n*=)	Performance Success Rate (%)			
Banfai et al., 2017 [27]	582	70,96	582	52,92			
Dhansura et al., 2020 [28]	132	97,73	132	67,42			
Meissner et al., 2012 [26]	132	92	98	95,9			
Naqvi et al., 2011 [29]	30	73,33	30	80			
Pooled Weighted Results	**876**	**78**,**25**	**842**	**61**,**16**		**1.8593**	**0.1599**

Study	Immediately After		2–4 Months After			t	*p*
	Total Count (*n*=)	Performance Success Rate (%)	Total Count (*n*=)	Performance Success Rate (%)			
Banfai et al. 2017 [27]	582	70,96	582	52,92			
Dhansura et al. 2020 [28]	132	97,73	132	67,42			
Meissner et al. 2012 [26]	132	92	98	95,9			
Naqvi et al. 2011 [29]	30	73,33	30	80			
Pooled Weighted Results	**876**	**78**,**25**	**842**	**61**,**16**		**1.8593**	**0.1599**

**Table 6:** Changes in rates of calling EMS in NRSIs immediately after vs. 6–8 months after training

Study	Immediately After		6–8 Months After		t	*p*
	Total Count (*n*=)	Performance Success Rate (%)	Total Count (*n*=)	Performance Success Rate (%)		
Cons-Ferreiro et al. 2023 [31]	260	85,00	91	92,30		
Müller et al. 2014 [30]	2579	91,20	1700	90,80		
Pooled Weighted Results	**2839**	**90**,**63**	**1791**	**90**,**88**	**−0.03793**	**0.9759**

#### Correct hand positioning

##### Overall quality of data

Three RCTs examined short-term outcomes but were limited by a lack of blinding, thus introducing performance bias [19, 22, 23]. The report by Sabihah et al. had a high risk of bias due to missing outcome data [19]. For long-term outcomes, data stems from three studies with lower risk of bias scores [17, 18, 22]. The two NRSIs assessing short-term outcomes showed a moderate overall risk of bias, primarily because of confounding factors, limitations in outcome measurement, and selective reporting [27, 29].

##### Outcome accomplishment over time

**Fig. 8:**
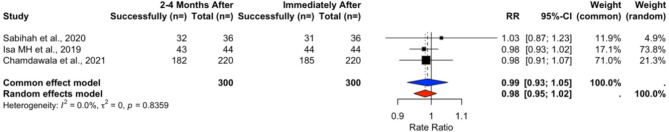
Rate ratio analysis of correct hand positioning in RCTs immediately after vs. 2–4 months after training

**Fig. 9:**
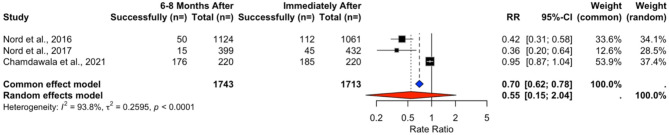
Rate ratio analysis of correct hand positioning in RCTs immediately after vs. 6–8 months after training

The pooled weighted success rates did not change significantly over time in the included RCTs, with a trend toward a decline, from 19.96% to 13.83% (t = 2.3585, *p* = 0.1424) at 6–8 months (Tables 7 and 8). In the absence of notable heterogeneity, we used a common effects model for RR analyses and found no significant differences over time (RR = 0.9884, 95% CI [0.9275, 1.0534], *p* = 0.7203) (Fig. 8). Changes over longer follow-up periods were assessed using a random-effects model revealing no significant changes over time (RR = 0.5468, 95% CI [0.1467, 2.0381], *p* = 0.1871) (Fig. 9).

**Table 7:** Changes in success rates of correct hand positioning in RCTs immediately after vs. 2–4 months after training

Study	Immediately After		2–4 Months After		t	*p*
	Total Count (*n*=)	Performance Success Rate (%)	Total Count (*n*=)	Performance Success Rate (%)		
Sabihah et al. 2020 [19]	36	86,11	36	88,89		
Isa MH et al. 2019 [23]	44	100	44	97,7		
Chamdawala et al. 2021 [22]	220	84,09	220	82,73		
Pooled Weighted Results	**300**	**86**,**67**	**300**	**85**,**66**	**0.8685**	**0.4767**

**Table 8:** Changes in success rates of correct hand positioning in RCTs immediately after vs. 6–8 months after training

Study	Immediately After		6–8 Months After		t	*p*
	Total Count (*n*=)	Performance Success Rate (%)	Total Count (*n*=)	Performance Success Rate (%)		
Nord et al. 2016 [17]	1061	10,55	1124	4,45		
Nord et al. 2017 [18]	432	10,41	399	3,76		
Chamdawala et al. 2021 [22]	220	84,09	220	80		
Pooled Weighted Results	**1713**	**19**,**96**	**1743**	**13**,**83**	**2.3585**	**0.1424**

**Fig. 10:**
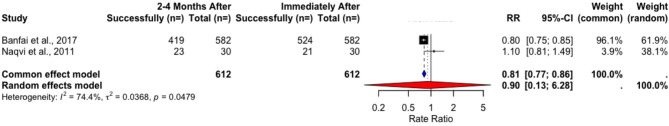
Rate ratio analysis of correct hand positioning in NRSIs immediately after vs. 2–4 months after training

In NRSIs, the pooled weighted success rates decreased from 89.02% to 72.23% (Table 9). However, neither the t-test (t = 0.96251, *p* = 0.5122), nor the random-effects model indicated any significant changes (RR = 0.9014, 95% CI [0.1294, 6.2798], *p* = 0.6201) (Fig. 10).

**Table 9:** Changes in success rates of correct hand positioning in NRSIs immediately after vs. 2–4 months after training

Study	Immediately After		2–4 Months After		t	*p*
	Total Count (*n*=)	Performance Success Rate (%)	Total Count (*n*=)	Performance Success Rate (%)		
Banfai et al. [27]	582	90	582	72		
Naqvi et al. [29]	30	70	30	76,67		
Pooled Weighted Results	**612**	**89**,**02**	**612**	**72**,**23**	**0.96251**	**0.5122**

#### Correct chest compression

##### Overall quality of data

The RCTs investigating short-term outcomes had a low to moderate risk of bias (except performance bias) [19, 22, 23]. This was also true for RCTs examining longer-term outcomes [17, 18, 22, 25] with the exception of two studies with higher risk [16, 24]. Among the NRSIs, Meissner et al. [26] demonstrated the lowest risk of bias with moderate concerns regarding confounding. Other studies had a moderate to high risk due to additional concerns associated with outcome measurement, selective reporting, and missing data [27, 29].

##### Outcome accomplishment over time

**Fig. 11:**
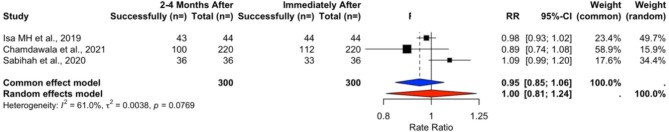
Rate ratio analysis of overall correct chest compressions in RCTs immediately after vs. 2–4 months after training

**Fig. 12:**
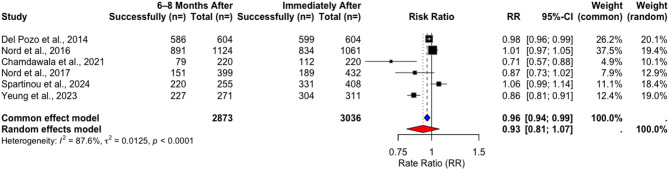
Rate ratio analysis of overall correct chest compressions in RCTs immediately after vs. 6–8 months after training

In the included RCTs, the weighted mean success rate immediately after training was 63.1% and decreased insignificantly to59.7% at 2–4 months post-training (t = 0.76002, *p* = 0.5266) (Table 10). In studies with longer follow-ups, the weighted success rate slightly declined from 78.08% to 75% after 6–8 months, again without a statistically significant difference (t = 0.51983, *p* = 0.6254) (Table 11). Similarly, rate ratio analyses using random-effects models indicated non-significant changes (RR = 1.0003, CI = [0.8097, 1.2357], *p* = 0.9962) after 2–4 months (Fig. 11) and after 6–8 months (RR = 0.9280, CI = [0.8069; 1.0672], *p* = 0. 2275) (Fig. 12).

**Table 10:** Changes in success rates of overall correct chest compressions in RCTs immediately after vs. 2–4 months after training

Study	Immediately After		2–4 Months After		t	*p*
	Total Count (*n*=)	Performance Success Rate (%)	Total Count (*n*=)	Performance Success Rate (%)		
Isa MH et al. 2019 [23]	44	100	44	97,7		
Chamdawala et al. 2021 [22]	220	51	220	45,5		
Sabihah et al. 2020 [19]	36	92	36	100		
Pooled Weighted Results	**300**	**63**,**11**	**300**	**59**,**70**	**0.76002**	**0.5266**

**Table 11:** Changes in success rates of overall correct chest compressions in RCTs immediately after vs. 6–8 months after training

Study	Immediately After		6–8 Months After		t	*p*
	Total Count (*n*=)	Performance Success Rate (%)	Total Count (*n*=)	Performance Success Rate (%)		
del Pozo et al. 2014 [16]	604	99,2	604	97		
Nord et al. 2016 [17]	1061	78,6	1124	79,3		
Chamdawala et al. 2021 [22]	220	50,9	220	36		
Nord et al. 2017 [18]	432	43,7	399	37,8		
Spartinou et al. 2024 [24]	408	81,12	255	86,27		
Yeung et al. 2023 [25]	311	97,74	271	83,92		
Pooled Weighted Results	**3036**	**78**,**02**	**2873**	**75**,**00**	**0.51983**	**0.6254**

**Fig. 13:**
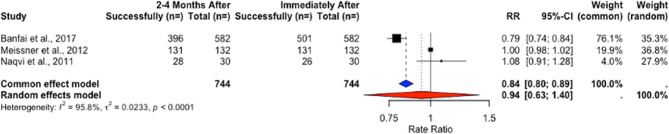
Rate ratio analysis of overall correct chest compressions in NRSIs immediately after vs. 2–4 months after training

In NRSIs, a decrease in weighted success rates from 88.17% immediately after training to 72.9% 2–4 months after training was observed (Table 12). However, neither the paired t-test (t = 1.4717, *p* = 0.2789) nor the random-effects model (RR = 0.9445, 95% CI [0.6246–1.4282], *p* = 0.6125) indicated any significant change (Fig. 13).

**Table 12:** Changes in success rates of overall correct chest compressions in NRSIs immediately after vs. 2–4 months after training

Study	Immediately After		2–4 Months After		t	*p*
	Total Count (*n*=)	Performance Success Rate (%)	Total Count (*n*=)	Performance Success Rate (%)		
Banfai et al. [27]	582	86,08	582	68,04		
Meissner et al. [26]	132	99,24	132	99,24		
Naqvi et al. [29]	30	86,67	30	93,33		
Pooled Weighted Results	**744**	**88**,**17**	**744**	**72**,**9**	**1.4717**	**0.2789**

#### Mean chest compression depth

In contrast to other outcomes, for which data were available and could be extracted from reports on various study designs, the analysis and reporting of this outcome are exclusively based on data from RCTs.

##### Overall quality of data

The risk of selection bias was small due to well-documented processes of random sequence generation and allocation concealment, but some studies exhibited points of concern regarding deviations from the intended intervention, [14, 15, 21, 22], missing outcome data, and measurement of the outcome [17, 20–22].

##### Changes for mean chest compression depth

Analyses of weighted mean depths using paired t-tests indicated non-significant changes in both short-term (Table 13) and long-term follow-ups (Table 14).

**Table 13:** Changes in compression depth in RCTs immediately after vs. 2–4 months after training

Study	Immediately After		2–4 Months After		t	*p*
	Total Count (*n*=)	Performance Success Rate (mm)	Total Count (*n*=)	Performance Success Rate (mm)		
Beskind et al. 2016 [14]	159	33,06	159	32,62		
Cuijpers et al. 2016 [15]	144	36,4	144	36,3		
Yeung et al. 2017 [21]	81	35,7	81	33,6		
Chamdawala et al. 2021 [22]	220	45,5	220	43,5		
Pooled Weighted Results	**604**	**38**,**74**	**604**	**37**,**59**	**1.838**	**0.1634**

**Table 14:** Changes in compression depth in RCTs immediately after vs. 6–8 months after training

Study	Immediately After		6–8 Months After		t	*p*
	Total Count (*n*=)	Performance Success Rate (mm)	Total Count (*n*=)	Performance Success Rate (mm)		
Nord et al. 2016 [17]	1061	40	1124	45		
Van Raemdonck et al. 2014 [20]	165	40,6	165	38,2		
Yeung et al. 2017 [21]	81	35,7	81	35,9		
Chamdawala et al. 2021 [22]	220	45,5	220	41		
Pooled Weighted Results	**1527**	**40**,**63**	**1590**	**43**,**28**	**−0.58526**	**0.5995**

#### Correct chest compression depth

##### Overall quality of data

Three RCTs demonstrated a low risk of bias across most domains [17, 18, 25]. While two other trials had high risk due to deviations from the intended intervention and missing outcome data [20, 24]. Included NRSIs [28, 29] had moderate risk of bias for this outcome, especially regarding its measurement [27].

##### Outcome accomplishment over time

**Fig. 14:**
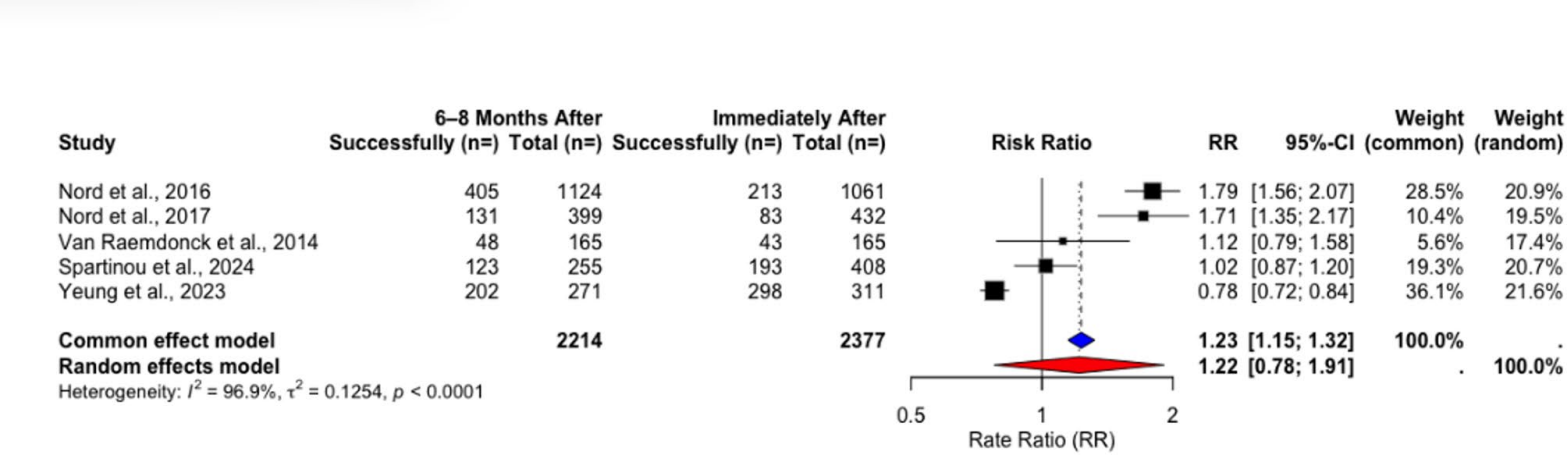
Rate ratio analysis of correct chest compression depth in RCTs immediately after vs. 6–8 months after training

Using data from the included RCTs, paired t-test (t = −0.53972, *p* = 0.618) and rate ratio analyses using a random-effects model indicated no significant changes over time (RR = 1.2162, 95% CI [0.7757; 1.9070], *p* = 0. 0.2934) (Table 15) (Fig. 14).

**Table 15:** Changes in success rates of correct compression depth in RCTs immediately after vs. 6–8 months after training

Study	Immediately After		6–8 Months After		t	*p*
	Total Count (*n*=)	Performance Success Rate (%)	Total Count (*n*=)	Performance Success Rate (%)		
Nord et al. 2016 [17]	1061	20,08	1124	36,03		
Nord et al. 2017 [18]	432	19,21	399	32,83		
Van Raemdonck et al. 2014 [20]	165	26,06	165	29,09		
Spartinou et al. 2024 [24]	408	47,3	255	48,23		
Yeung et al. 2023 [25]	311	95,81	271	74,59		
Pooled Weighted Results	**2377**	**34**,**92**	**2214**	**41**,**06**	**−0.53972**	**0.618**

**Fig. 15:**
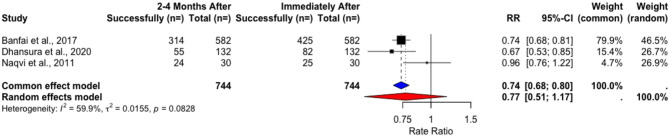
Rate ratio analysis of correct chest compression depth in NRSIs immediately after vs. 2–4 months after training

**Fig. 16:**
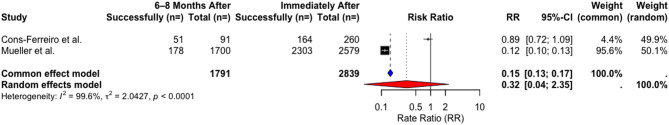
Rate ratio analysis of correct chest compression depth in NRSIs immediately after vs. 6–8 months after training

The pooled success rates in NRSIs changed within the short follow-up of 2–4 months (71.5% to 52.8%) but these changes were not significant in t-tests (t = 1.3958, *p* = 0.2971) (Table 16) and the random-effects model (RR = 0.7725, 95% CI [0.5083, 1.1739], *p* = 0.1175) (Fig. 15). A large change in success rates was observed in long-term follow-ups, from 86.9% to 12.81% after 6–8 months (Table 17), but these results were not significant in formal testing due to heterogeneity (RR = 0.3223, 95% CI [0.0443; 2.3453], *p* = 0.263) (Fig. 16).

**Table 16:** Changes in success rates of correct compression depth in NRSIs immediately after vs. 2–4 months after training

Study	Immediately After		2–4 Months After		t	*p*
	Total Count (*n*=)	Performance Success Rate (%)	Total Count (*n*=)	Performance Success Rate (%)		
Banfai et al. 2017 [27]	582	73	582	54		
Dhansura et al. 2020 [28]	132	62,12	132	41,66		
Naqvi et al. 2011 [29]	30	83,33	30	80		
Pooled Weighted Results	**744**	**71**,**49**	**744**	**52**,**86**	**1.3975**	**0.2971**

**Table 17:** Changes in success rates of correct compression in depth in NRSIs immediately after vs. 6–8 months after training

Study	Immediately After		6–8 Months After		t	*p*
	Total Count (*n*=)	Performance Success Rate (%)	Total Count (*n*=)	Performance Success Rate (%)		
Cons-Ferreiro et al. 2023 [31]	260	63,07	91	56,04		
Müller et al. 2014 [30]	2579	89,3	1700	10,5		
Pooled Weighted Results	**2839**	**86**,**90**	**1791**	**12**,**81**	**1.0859**	**0.4738**

#### Mean chest compression rate

Analysis and reporting of this outcome is solely based on data from RCTs since mean chest compression rate was not reported in the other studies.

##### Overall quality of data

In the studies assessing short-term effects, random sequence generation and allocation concealment were largely conducted with low likelihood of selection bias. However, risk of performance and detection biases was detected in most of the included studies, ultimately leading to an overall moderate risk of bias concerning this outcome [14, 15, 21, 22]. This was also true for studies evaluating long-term outcomes [17, 20–22].

##### Changes in mean compression rate

The overall weighted mean compression rates changed non-significantly, from 106.08 to 105.01 compressions per minute after 2–4 months, and from 108.75 to 102.1 compressions per minute after 6–8 months. Paired t-tests revealed no statistically significant difference (t = 0.61789, *p* = 0.5804 and t = 1.6181, *p* = 0.2041 respectively) (Tables 18 and 19).

**Table 18:** Changes in compression rate in RCTs immediately after vs. 2–4 months after training

Study	Immediately After		2–4 Months After		t	*p*
	Total Count (*n*=)	Performance Success Rate (/bpm)	Total Count (*n*=)	Performance Success Rate (/bpm)		
Beskind et al. 2016 [14]	159	99,1	159	98,1		
Cuijpers et al. 2016 [15]	144	98	144	101,5		
Yeung et al. 2017 [21]	81	119,4	81	109,9		
Chamdawala et al. 2021 [22]	220	111,5	220	110,5		
Pooled Weighted Results	**604**	**106**,**08**	**604**	**105**,**01**	**0.61789**	**0.5804**

**Table 19:** Changes in compression rate in RCTs immediately after vs. 6–8 months after training

Study	Immediately After		6–8 Months After		t	*p*
	Total Count (*n*=)	Performance Success Rate (/bpm)	Total Count (*n*=)	Performance Success Rate (/bpm)		
Nord et al. 2016 [17]	1061	111,5	1124	102		
Van Raemdonck et al. 2014 [20]	165	92,6	165	87,8		
Yeung et al. 2017 [21]	81	119,4	81	109,5		
Chamdawala et al. 2021 [22]	220	111,5	220	109		
Pooled Weighted Results	**1527**	**109**,**88**	**1590**	**101**,**88**	**−1.6181**	**0.2041**

#### Correct chest compression rate

##### Overall quality of data

Four RCTs reported long-term performance with two reports having low risk of bias in most domains except for performance bias, and two reports having high risk of bias due to outcome classification and missing data [17, 20, 24, 25].

Five NRSIs assessed short-term outcomes. Of these, three exhibited a generally low risk of bias across most domains [27–29], and two had moderate to high risk due to issues concerning missing data [30, 31].

##### Outcome accomplishment over time

**Fig. 17:**
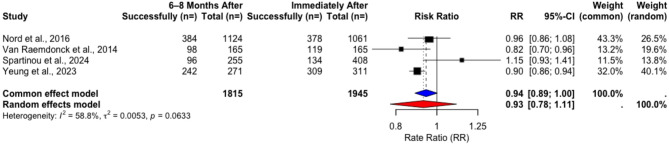
Rate ratio analysis of correct chest compression rate in RCTs immediately after vs. 6–8 months after training

After 6–8 months, data from RCTs showed a decrease of the pooled success rates from 48.33% to 45.17%. with paired t-tests (t = 0.86117, *p* = 0.4525) (Table 20) and RR analyses showing non-significant difference (RR = 0.9432, 95% CI [0.8871; 1.0029], *p* = 0.0618) (Fig. 17).

**Table 20:** Changes in success rates of correct compression rate in RCTs immediately after vs. 6–8 months after training

Study	Immediately After		6–8 Months After		t	*p*
	Total Count (*n*=)	Performance Success Rate (%)	Total Count (*n*=)	Performance Success Rate (%)		
Nord et al. 2016 [17]	1061	35,63	1124	34,16		
Van Raemdonck et al. 2014 [20]	165	72,12	165	59,39		
Spartinou et al. 2024 [24]	408	32,84	255	37,64		
Yeung et al. 2023 [25]	311	99,35	271	89,29		
Pooled Weighted Results	**1945**	**48**,**33**	**1815**	**45**,**17**	**0.86117**	**0.4525**

**Fig. 18:**
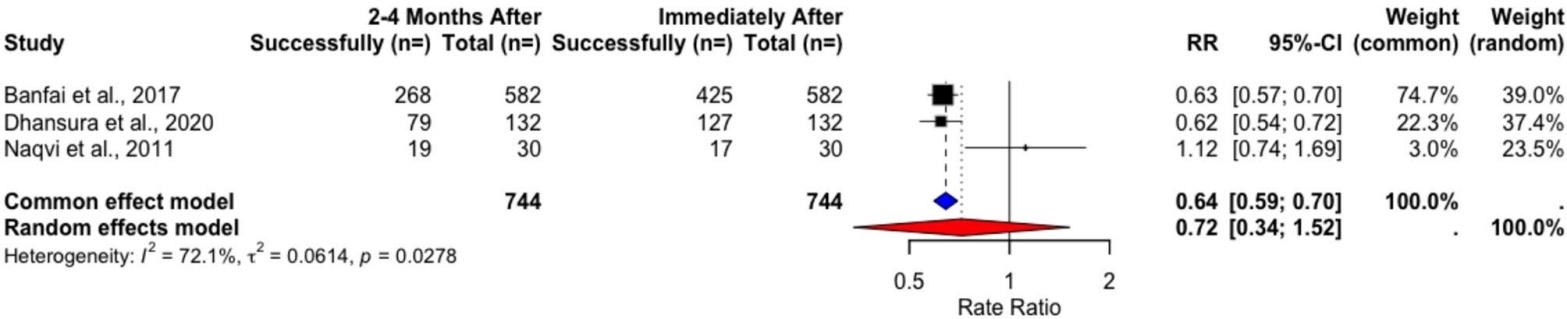
Rate ratio analysis of correct chest compression rate in NRSIs immediately after vs. 2–4 months after training

**Fig. 19:**
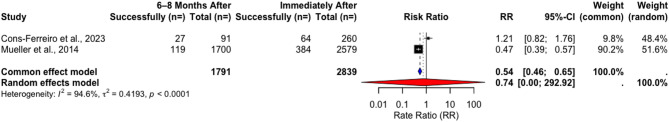
Rate ratio analysis of correct chest compression rate in NRSIs immediately after vs. 6–8 months after training

In NRSIs, the weighted success rate results decreased after 2–4 months (76.46% to 49.16%), but neither the paired t-test (t = 1.4411, *p* = 0.2863) nor RR analysis using a random-effects model showed a statistically significant decline (RR = 0.7178, 95% CI [0.3399, 1.5159], *p* = 0.1966) (Table 21) (Fig. 18). The long-term follow-up showed changes in success rates from 15.79% to 8.15% (t = 1.2429, *P* = 0.4314) (Table 22), but again, RR analysis under a random-effects model showed no statistical significance (RR = 0.7417, 95% CI [0.0019; 292.9188], *p* = 0.6399) (Fig. 19).

**Table 21:** Changes in success rates of correct compression rate in NRSIs immediately after vs. 2–4 months after training

Study	Immediately After		2–4 Months After		t	*p*
	Total Count (*n*=)	Performance Success Rate (%)	Total Count (*n*=)	Performance Success Rate (%)		
Banfai et al. 2017 [27]	582	73	582	46		
Dhansura et al. 2020 [28]	132	96,21	132	59,85		
Naqvi et al. 2011 [29]	30	56,67	30	63,33		
Pooled Weighted Results	**744**	**76**,**46**	**744**	**49**,**16**	**1.4411**	**0.2863**

**Table 22:** Changes in success rates of correct compression rate in NRSIs immediately after vs. 6–8 months after training

Study	Immediately After		6–8 Months After		t	*p*
	Total Count (*n*=)	Performance Success Rate (%)	Total Count (*n*=)	Performance Success Rate (%)		
Cons-Ferreiro et al. 2023 [31]	260	24,61	91	29,67		
Müller et al. 2014 [30]	2579	14,90	1700	7,00		
Pooled Weighted Results	**2839**	**15**,**79**	**1791**	**8**,**15**	**1.2429**	**0.4313**

## Discussion

This systematic review and meta-analysis assessed the effectiveness of school-based CPR training and the retention of CPR skills over time. A total of twelve RCTs and six NRSIs were included in the meta-analysis, which assessed both the immediate effects of training and the long-term durability of skills at multiple follow-up points. With regard to immediate training outcomes, generally referred to as effectiveness, data indicate that trainings were indeed effective in allowing children to attain a certain level of CPR proficiency. This was unsurprising and had been demonstrated in previous reviews, albeit without formal meta-analysis [32]. What remained to be investigated was the extent to which any decline in skills occurred over time. In this regard, our analysis revealed a time-dependent tendency towards a decline in CPR skill retention. Although initial training yielded high success rates, skills such as calling EMS and correct hand positioning deteriorated in the months after training. Results of NRSIs demonstrated a greater decline in CPR skills compared to RCTs. While these declines were not statistically significant in formal analyses, the trends were stable across multiple outcomes, and more pronounced after longer follow-up times.

Overall, changes in CPR-related skills were small in the short term, and skills were retained for at least 2–4 months. As time went on, this changed slightly, and the status after 6–8 months was less clear. Data from most studies suggested at least some relevant decline in proficiency. This finding is in line with results of previous studies [33, 34]. The need for CPR training may thus need to be viewed as an ongoing process rather than a one-time event. Periodic refresher courses or booster sessions could be essential to ensure long-term retention of critical skills and would likely be most efficient if integrated into school curricula.

### Limitations

The review has several limitations. First, there was substantial heterogeneity among the included studies in terms of methodology, sample size, training formats, and assessment tools. Although both fixed- and random-effects models were applied in the meta-analysis to account for this variability, such methodological differences likely introduced residual heterogeneity that may have influenced the pooled results. Second, the overall quality and completeness of data varied. Several studies lacked essential statistical measures – such as standard deviations or confidence intervals – limiting the ability to compute pooled effect sizes. Moreover, discrepancies in follow-up intervals across studies complicated comparisons of skill retention over time and may have obscured true patterns of decay or persistence.

Third, in some studies, multiple CPR training components or delivery methods were combined within the same intervention arm, making it difficult to disentangle the individual effects of specific modalities (e.g., instructor-led vs. video-based training). This limits the precision of conclusions regarding which training formats are most effective for skill acquisition or retention. Additionally, most included studies primarily focused on technical skill performance, with limited attention to important secondary outcomes such as participants’ confidence, self-efficacy, and willingness to perform CPR in real-life scenarios. These psychological and behavioral aspects are crucial determinants of actual bystander intervention but remain underexplored in the current literature. The scarcity of data on these factors represents a significant knowledge gap and highlights the need for future research to adopt a more holistic evaluation framework. Finally, potential publication bias cannot be excluded, as studies with positive findings are more likely to be published. This may have led to an overestimation of the effectiveness of school-based CPR training.

#### Implications for future research and practice

School-based CPR training not only equips students with life-saving skills but may also promote broader health awareness and emergency preparedness, with potential ripple effects extending to families and communities [35, 36]. In addition, such training has been associated with increased confidence and a greater willingness to intervene during emergencies, even if the concrete effects on real-life scenarios remain unclear [37, 38]. However, few of the studies included in this review assessed these psychological and behavioral outcomes directly. Future research should explore these dimensions to better understand the comprehensive impact of CPR education on readiness to respond in real-life scenarios.

##### Reinforcement training

Evidence from this review supports the inclusion of booster or refresher sessions to counteract skill decay. These follow-ups could help maintain the accuracy and quality of CPR performance over time and should be considered a standard component of school-based programs.

##### Standardization and methodological improvements

The heterogeneity in follow-up intervals, outcome measures, and assessment tools across studies complicates direct comparison and limits the generalizability of findings. Similar issues have been noted in evaluations of first aid training, where inconsistent assessment methods impede the synthesis of broad conclusions [39]. These challenges highlight the need for standardized protocols in future CPR research, including uniform timing of follow-up assessments and consistent evaluation criteria for both technical and non-technical outcomes.

##### Standardized metrics and long-term evaluation

The adoption of standardized metrics to not only evaluate CPR quality such as compression depth, rate, and hand positioning, but also aspects like participants’ willingness to perform CPR and non-CPR related outcomes with potential health effects, would enhance the reliability of findings and facilitate meaningful comparisons across studies. Moreover, research should aim to determine the optimal training duration and instructional content needed for long-term skill retention. Evaluating the effectiveness of various delivery methods, including hands-on practice, digital simulations, and hybrid formats, is also warranted. Finally, longitudinal studies extending beyond the commonly used 6–8-month follow-up period are essential. Such research would provide deeper insight into the natural trajectory of skill retention and decay, and help identify the most effective strategies for sustaining competence over time.

## Conclusion

While school-based CPR training effectively teaches children CPR skills, these skills may deteriorate over time, suggesting a need for periodic refresher courses to sustain proficiency. Standardized evaluation methods and reinforcement strategies should be incorporated into training programs to ensure sustained competence and maximize educational outcomes.

## Supplementary Information

Supplementary Material 1

## Data Availability

All data used in the analysis are publicly available.

## Abbreviations

CI: Confidence interval
CPR: Cardiopulmonary resuscitation
EMS: Emergency medical services
NRSI: Non-randomized studies of interventions
RCT: Randomized controlled trials
RR: Rate ratio
